# Reliability of the Nepali Version of the Spinal Cord Independence Measure Self-Report

**DOI:** 10.1155/2022/9983464

**Published:** 2022-06-09

**Authors:** Prakriti Khatri, Saipin Prasertsukdee, Jatuporn Suttiwong

**Affiliations:** Faculty of Physical Therapy, Mahidol University, Salaya, Thailand

## Abstract

A self-report measure is considered a practical alternative tool for longitudinal monitoring and for community models of disability. Spinal Cord Independence Measure Self-Report (SCIM-SR) was developed to measure the functional independence of the spinal cord injury (SCI) population. For the application of this questionnaire in Nepali setting, the cross-cultural adaptation and reliability of the Nepali version of the SCIM-SR were also warranted. The aim of the study was to cross-culturally adapt the Nepali version of the SCIM-SR and determine its reliability. The English version of the SCIM-SR was translated into the Nepali language with cross-cultural adaptations following the Beaton guidelines. A total of 45 community-dwelling individuals with SCI administered the Nepali version of the SCIM-SR two times, with an interval of one week. The intraclass correlation coefficient (ICC) and Cronbach's alpha (*α*) were used to assess the test-retest reliability and internal consistency, respectively. Cross-cultural equivalence was achieved between the English version and the Nepali version of the SCIM-SR. The test-retest reliability was excellent, with ICCs for the total score 0.968 (95% confidence interval 0.941–0.982), self-care subscale 0.964 (0.934–0.980), respiration and sphincter management subscale 0.941 (0.893–0.968), and mobility subscale 0.966 (0.938–0.981). The internal consistency reached an acceptable range for the total score and most of the subscales except for those of respiration and sphincter management. Cronbach's *α* coefficients for the total score, self-care subscale, respiration and sphincter management subscale, and mobility subscale were 0.801, 0.959, 0.506, and 0.838, respectively. The Nepali version of the SCIM-SR was cross-culturally adapted and can be used as a reliable self-report instrument to assess functional independence among the community-dwelling SCI population in Nepal.

## 1. Introduction

Spinal cord injury (SCI) leads to impairments in several body structures and functions, limitations in daily life activities, and restrictions in participation in social life [1, 2]. The SCI rehabilitation of those individuals from acute care to community life leads to an increase in their functional level [3]. Assessments of functional outcomes are an integral part of SCI rehabilitation. These assessments help the interdisciplinary team inform patients and their caregivers about prognosis, plan the rehabilitation process, and predict long-term functional outcomes [4]. Hence, the use of reliable and valid outcome measurement tools is considered important for good clinical practice and evidence-based health care [5]. The Spinal Cord Independence Measure (SCIM) is a disability scale which assesses activities of daily living relevant to the SCI population [6]. However, it is a clinician-based instrument and can be applied mainly in the inpatient setting [7].

The development and use of health-related self-report outcome measures, along with the establishment of their psychometric properties, have increased concomitantly with the popularity of a patient-centered treatment approach [8]. These questionnaires are considered important because they detect special health care needs from the patient's perspective and are used for long-term follow-up and community-based rehabilitation models. Moreover, they require relatively few health care resources and efforts for administration than the clinician-rated instrument and provide fast data collection [7, 9].

The Spinal Cord Independence Measure Self Report (SCIM-SR) is a patient-reported instrument which assesses the ability of individuals with SCI to perform everyday activities independently [7]. The SCIM-SR comprises 3 subscales: self-care, respiration and sphincter management, and mobility. The total score ranges from 0 to 100, with a higher score reflecting more independence. The SCIM-SR was developed in the English language, based on the English version of SCIM III [7]. It has been translated and validated into various languages: German, Italian, Spanish, Portuguese, Thai, Chinese, Japanese, and Swedish. Previous studies have shown the SCIM-SR to be a reliable and valid instrument to measure functional outcomes among SCI population [7, 10–16].

In Nepal, assessment of the functional outcomes among people with SCI after hospital discharge has been overlooked in the rehabilitation area. The manifestation of long-term complications deteriorates their functional abilities, further increases their disability, and hence significantly decreases their quality of life [17, 18]. Lifetime follow-ups are needed to meet their changing health care needs and to prevent secondary complications [19]. Due to the lack of resources in the community of Nepal, self-report questionnaires are promising tools for incorporating the patient's perspectives into clinical care and research. Although the SCIM-SR is a comprehensive tool and has good psychometric properties, its application in the Nepali SCI population is currently limited. A culturally relevant tool is needed by the SCI rehabilitation team to assess functional status from acute to long-term care. To date, there is no evidence of functional outcome measures in Nepali culture for the SCI population. Thus, the present study aimed to cross-culturally adapt the Nepali version of the SCIM-SR and determine its reliability.

## 2. Materials and Methods

This is a methodological study. The study was approved by Mahidol University-Central Institutional Review Board, Thailand with Reg. No. 2020/209.1708 and Nepal Health Research Council Reg. No. 692/2020 MT. The use of the SCIM-SR was authorized by the developer of the questionnaire. Individuals with SCI were included based on purposive sampling from December 2020 to February 2021. The participants were recruited from the communities of Nepal and those with outpatient follow-up appointments at the Spinal Injury Rehabilitation Centre (SIRC), Nepal. For inclusion, all participants were required to meet the following criteria: aged between 18 and 60 years, traumatic or non-traumatic SCI with the time of onset of injury more than one month, neurological classification of SCI (American Spinal Injury Association Impairment Scale (AIS)–A, B, C, D), and able to read and write the Nepali language. Participants were excluded if they had other concomitant injuries(e.g., traumatic brain injury or fractures), and were currently involved in active inpatient rehabilitation. Additionally, participants who reported a change in their functional status within a week after the first administration of the questionnaire were also excluded. The sample size was 45 when the alpha was set at 0.05, and the power was 80% to detect a value of 0.40 for the intraclass correlation (ICC) test with 20% dropouts [20].

### 2.1. Procedures

This study comprised two phases; the translation and cross-cultural adaptation of the SCIM-SR and the reliability testing of the Nepali version of the SCIM-SR.

### 2.2. Phase I Translation and Cross-cultural adaptation of the SCIM-SR

**Figure 1:**
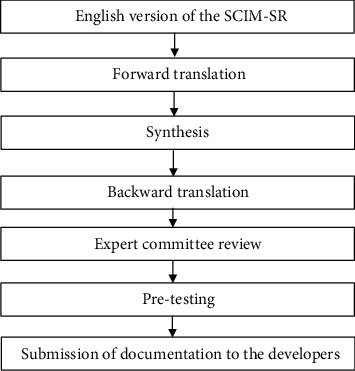
Translation and cross-cultural adaptation of the Nepali version of the SCIM-SR (Beaton guidelines)

The translation and cross-cultural adaptation of the SCIM-SR were performed following Beaton's guidelines, which include the following steps: forward translation, synthesis, backward translation, expert committee meeting, cognitive testing, and submission to the developer as shown in Figure 1 [21]. Two forward translators, namely, one medical (MSc. in Physical Therapy) and non-medical (MA in Nepali), who were bilingual and spoke Nepali as their mother language, translated the English version of the SCIM-SR into the Nepali language. One common translation was produced from two forward translations. The language discrepancies were noted and solved by consensus. Then, two backward translations were produced by two native English speakers who were non-medical persons (MA in intercultural studies), capable in both the spoken and written Nepali language, and not informed about the concepts explored. The expert committee included one physical therapist with >2 years of experience in SCI rehabilitation, one physiatrist with >2 years of experience in SCI rehabilitation, one methodologist with experience in translating the Nepali language, forward translators, and backward translators. Certain items were modified to match the cultural preferences and daily activities of Nepalese people. The committee members considered semantic, idiomatic, experiential, and conceptual equivalence between the English version and the Nepali version of the SCIM-SR.

- Semantic equivalence: Do the words in the translated version of the Nepali SCIM-SR mean the same thing in the English version? The words that are difficult to understand by SCI population of Nepal, such as medical terminologies, were noted and adjusted by giving explanations. For example, in item 1, eating and drinking “stomach tube” was adjusted by adding an explanation as “a tube which is inserted into stomach for feeding purpose”.
- Idiomatic equivalence: Does the questionnaire have idioms that are difficult to translate? The committee aimed to formulate an equivalent expression in the Nepali version.
- Experiential equivalence: Does the questionnaire have items that capture experience of daily life in the Nepali culture? These items were replaced by similar items that is in fact experienced in Nepali culture. For example, kurta, a common dress used by women in Nepal, was added in item 3A dressing your upper body.
- Conceptual equivalence: Does the questionnaire have items that hold similar conceptual meanings between cultures?

After that, they developed the prefinal version. Cognitive interviews of the preliminary draft were performed among five community-dwelling individuals with SCI [7]. Finally, the report was submitted to the developer of the SCIM-SR.

### 2.3. Phase II Reliability Testing

Sociodemographic (i.e., age, gender, education level, ethnicity) and neurological details, such as age at injury, duration of injury, cause of injury, level of injury, and AIS grade, were recorded from medical files and via interviews. The participants were screened according to selection criteria, and informed consent was obtained. Then, the participants were asked to complete the Nepali version of the SCIM-SR via paper-pencil version in person or online form via e-mail. After one week, they were asked to readminister this self-report questionnaire [22]. The participants were assisted in filling out the questionnaire if they had difficulty writing owing to limited hand function. However, the items were not explained, and they were not assisted in choosing an answer.

### 2.4. Data Analysis

All statistical evaluations were performed using Statistical Package for the Social Sciences (SPSS version 22; IBM, New York, USA). Descriptive statistics on the sample characteristics were expressed as mean, standard deviation or frequency, and percentage. Internal consistency was analyzed using Cronbach's alpha (*α*). Values of Cronbach's *α* exceeding 0.7 generally support reasonable internal consistency [23]. Test-retest reliability was determined using the intraclass correlation coefficient (ICC 3.1) with 95% confidence intervals between the initial and follow-up assessment. Scores of <0.50 were considered poor reliability, 0.50–0.75 were considered moderate, 0.75–0.90 were considered good, and >0.90 were considered excellent [24]. The level of significance was set at *p* value <0.05.

## 3. Results

A total of 45 individuals with SCI were recruited out of 47 participants who met the eligibility criteria. Two participants were excluded as they reported secondary complications resulting change in their functional status within the test-retest interval. The mean (SD) age of the participants was 29.6 (8.9) years, with ages ranging from 18 to 59 years. All participants had chronic SCI with a mean duration of injury of 8.1 (4.7) years, ranging from 1.5 to 17 years. The mean (SD) age at injury was 21.7 (8.9) years. The predominant causes of injury were falls (51.1%), followed by road traffic accidents. The cervical level of injury was 8 (17.8%), and that for the lumbar was 11 (24.4%), with thoracic injury being the most common 26 (57.8%). The demographic details of the participants are displayed in Table 1.

The Nepali version of the SCIM-SR was comprehensible as reported by the participants. Cognitive interviews with the participants, expert committee discussion, and the developer's feedback provided valuable insights into developing the final version of the questionnaire. Some medical terminologies, such as stomach tube, neck brace, corset, and indwelling catheter were adjusted by adding explanations to achieve semantic equivalence. Many items (2A, 2B, 3A, 6, 11, and 16) needed to adapt following the Nepali culture to achieve experimental equivalence between the English version and the Nepali version of the SCIM-SR as shown in Table 2.

During the cognitive interview, all respondents encountered difficulty in understanding the title. The term “Sushumna” means spinal cord in the Nepali language and is not commonly used by health professionals and the SCI population in Nepal. Therefore, it was translated into the Nepali language followed by transliteration. Moreover, the respondents reported that they were not familiar with PEEP and BIPAP in item 5 (breathing). They were independent in breathing and coughing and had never used these devices previously. However, the expert committee members (SCI rehabilitation team) had experienced the patients and caregivers using those words in case they have used them. Therefore, the expert committee members decided to keep the words “PEEP” and “BiPAP.” Additionally, the frequency of the bowel movements in item 7B was not clear. My bowel movements are irregular or seldom (less than once in 3 days) which were adjusted into “I pass stool irregular or seldom (not even once in 3 days).” My bowel movements are regular (once in 3 days or more) which were not clear about “more” in this context as the participants were concerned about diarrhea.

The Nepali version of the SCIM-SR demonstrated excellent test-retest reliability for total scores and all subscales, reflecting stability on repeated measures. The ICCs of the total score, self-care subscale, respiration and sphincter management subscale, and mobility subscale were 0.968, 0.964, 0.941, and 0.966, respectively, as shown in Table 3. The internal consistency reached an acceptable range for the total score and most of the subscales except for those of respiration and sphincter management. Cronbach's *α* coefficients for the total score, self-care subscale, and mobility subscale were 0.801, 0.959, and 0.838, respectively, as shown in Table 4. Cronbach's *α* coefficient was 0.506 for the respiration and sphincter management subscale. The Nepali version of the SCIM-SR provided consistency between items and the total questionnaire.

## 4. Discussion

The result supports the reliability of the Nepali version of the SCIM-SR. The ICCs of the total score, self-care subscale, and mobility subscale were consistent with those of the SCIM-SR in the Thai version, the Chinese version, and the Swedish version [12, 13, 16]. Previous studies have shown good to excellent test-retest reliability [12, 13, 16]. The test-retest reliability was highest in mobility subscale and lowest in respiration and sphincter management subscale in these studies, which is similar to the findings of this study. Moreover, the ICC of respiration and sphincter management subscale in this study was greater than that in previous studies [12, 13, 16]. In this study, the test-retest interval was one week to ensure that the functional status of the participants would not change and further exclusion of two participants who reported functional change affecting the reliability might have resulted in high test-retest reliability.

Cronbach's *α* values were above 0.7 for the total score, self-care, and mobility subscales, which are similar to those of the SCIM-SR in the Thai version, the Chinese version, the Japanese version, and the Swedish version [12, 13, 15, 16]. In this study, Cronbach's *α* for respiration and sphincter management subscale was 0.506, which was similar to that found in the Chinese version (*α* = 0.581), the Japanese version (*α* = 0.62), and the Swedish version (*α* = 0.37). Interestingly, the alpha values decreased in respiration and sphincter management subscale compared with the values of other subscales across all studies [12, 13, 15, 16].

Cronbach's *α* values are affected by item interrelatedness and by the number of test items. A lower number of items, poor interrelatedness between items, or heterogeneous constructs might result in low alpha values [25]. In this study, removal of item 5 breathing (*α* = 0.55) increased Cronbach's *α* of respiration and sphincter management subscale but elimination of other items: bladder management (*α* = 0.237), bowel management (*α* = 0.238), and using the toilet (*α* = 0.481) decreased this subscale Cronbach's *α*. The SCIM-SR was developed from the SCIM III which has also shown similar results in previous studies. The findings of the Rasch analysis of the SCIM III reported that respiration showed as a misfit, with a low correlation with the item of respiration and sphincter management subscale [26, 27]. Items such as bladder management, bowel management, and using the toilet fall under the urination and defecation functions, while breathing falls under a different domain. However, respiration was included along with bladder/bowel management and using the toilet subscale for scoring convenience. These items were scored by the same members of the multidisciplinary team. It has been reported that the internal consistency of the respiration and sphincter management subscale can be improved by adding more items that assess respiration-related disability, such as airflow and secretion clearance, or by creating separate subscales for respiration or bowel/bladder management/using the toilet when the SCIM III is revised [26–28].

Special attention should be given while filling item 6 bladder management. In item 6A if the patient uses indwelling catheter, item 6B intermittent catherization and item 6C use of external drainage instruments should not be filled. Scoring of items 6 and 7-Appendix B/C needs to be shifted at the end of the questionnaire as the participants encountered confusion during the cognitive testing. A separate sheet with the questionnaires should be given to the participants as scoring is only relevant for the health professionals.

The limitations of this study were that only individuals with SCI with chronic conditions were recruited. Therefore, the result might not be generalized to acute conditions. The Nepali version of the SCIM-SR is not applicable to the SCI population who cannot read the Nepali language. There are 124 castes and 123 languages in Nepal and only 45% of the Nepalese speak Nepali language. Additionally, interpretation of the findings of this self-report questionnaire should be done cautiously among the participants with cognitive deficits or among the older population.

## 5. Conclusions

The Nepali version of the SCIM-SR was cross-culturally adapted to the Nepali setting. The SCIM-SR demonstrated excellent test-retest reliability and acceptable internal consistency except respiration and sphincter management subscale. It is made available as a reliable instrument to assess functional independence among community-dwelling SCI population in clinical practice and research. It requires less healthcare resources and less time for its application, increasing its feasibility. Further exploration of psychometric properties such as validity and responsiveness may enhance the clinical utility of the Nepali version of the SCIM-SR in the Nepali setting.

## Figures and Tables

**Table 1:** Demographic and clinical characteristics of the participants (*n* = 45).

Items	Frequency (%)
Age	
18-30	29 (64.4)
31-45	13 (28.9)
46-60	3 (6.7)
Gender	
Male	37 (82.2)
Female	8 (17.8)
Type of disability	
Quadriplegia	8 (17.8)
Paraplegia	37 (82.2)
AIS grade	
A	35 (77.8)
B	6 (13.3)
C	4 (8.9)
Education level	
Uneducated/never been to the school	1 (2.2)
Primary education	4 (8.9)
Secondary education	6 (13.3)
Higher secondary level	23 (51.1)
Bachelor level	6 (13.3)
Masters or higher	5 (11.1)
Ethnicity	
Brahmin	11 (24.4)
Chhetri	11 (24.4)
Magar	7 (15.6)
Newar	5 (11.1)
Sherpa/Tamang	5 (11.1)
Tharu	2 (4.4)
Gurung	2 (4.4)
Thakuri	1 (2.2)
Rai/Limbu	1 (2.2)
Etiology	
Disease	2 (4.4)
Falls	23 (51.1)
Road traffic accident	14 (31.1)
Sports injuries	4 (8.9)
Disaster	2 (4.4)
Mode of administration	
Paper-pencil version	27 (60)
E-SCIM-SR	18 (40)
Assistance	
Assistance required	2 (4.4)
Assistance not required	43 (95.6)

*N*: number of participants; AIS: American Spinal Injury Association Impairment Scale.

**Table 2:** Cross-cultural adaptation of the Nepali version of the SCIM-SR.

Item no.	Source item	English version	Nepali version	Remarks
2A	Washing your upper body and head	Washing your upper body and head includes soaping and drying and using a water tap.	Washing your upper body and head includes activities like soaping, washing using a water tap, wiping, and drying.	Experiential equivalence The focus was made on the sequence and wiping and washing were added.

2B	Washing your lower body	Same as 2A	Same as 2A	Experiential equivalence

3A	Dressing your upper body	Dressing the upper body includes putting on and taking off clothes like t-shirts, blouses, shirts, bras, shawls, or orthoses (e.g., arm splint, neck brace, and corset).	Dressing the upper body includes putting on and taking off clothes like t-shirts, kurta, blouses, shirts, bras, shawls, ... (corset).	Experiential equivalence Kurta is a common dress for females in Nepal.

6	Bladder management	Use of external drainage instruments (e.g., condom catheter, diapers, and sanitary napkins)	Use of external drainage instruments (e.g., condom catheter, diapers, and pads/cotton clothes used during mensuration)	Experiential equivalence Cotton clothes that are commonly used by female during mensuration were added.

11	Transfer from the wheelchair to the toilet	Transfers from the wheelchair to the toilet/tub	Transfers from the wheelchair to the toilet/tub/shower chair	Experiential equivalence Shower chair and commode chair which are commonly used by people with disability in Nepal were added.
		Transferring also includes transfers from the wheelchair or bed to a toilet wheelchair.	Transferring also includes transfers from the wheelchair or bed to a toilet wheelchair/commode chair.	

16	Transfers from the wheelchair into the car	Transfers from the wheelchair into the car	Transfers from the wheelchair into the car/three-wheeled scooter	Experiential equivalence Three-wheeled scooter which is used by the SCI population in Nepal was added.

**Table 3:** Test-retest reliability of the Nepali version of the SCIM-SR.

Domains	ICC (3.1)	95% CI
Self-care subscale	0.964	0.934-0.980
Respiration and sphincter management subscale	0.941	0.893-0.968
Mobility subscale	0.966	0.938-0.981
Total score	0.968	0.941-0.982

ICC: intraclass correlation coefficient; CI: confidence interval.

**Table 4:** Internal consistency of the Nepali version of the SCIM-SR.

Items	Cronbach's alpha
*Self-care subscale*	0.959
Cronbach's alpha if item is deleted	
Item no.1: eating	0.948
Item no. 2A: washing your upper body and head	0.951
Item no. 2B: washing your lower body	0.954
Item no. 3A: dressing your upper body	0.957
Item no. 3B: dressing your lower body	0.949
Item no.4: grooming	0.950
*Respiration and sphincter management subscale*	0.506
Cronbach's alpha if item is deleted	
Item no.5 breathing	0.550
Item no.6 bladder management	0.237
Item no.7 bowel management	0.238
Item no.8 using the toilet	0.481
*Mobility subscale*	0.838
Cronbach's alpha if item is deleted	
Item no.9: mobility in bed and action to prevent pressure sores	0.850
Item no.10: transfer from bed to a wheelchair	0.829
Item no.11: transfer from wheelchair to toilet tub	0.829
Item no.12: moving around indoors	0.8
Item no.13: moving around moderate distances (10-100 meters)	0.796
Item no.14: moving around more than 100 meters	0.787
Item no.15: going up or downstairs	0.831
Item no.16: transfer from wheelchair to the car	0.825
Item no.17: transfer from floor to wheelchair	0.834
*Total score*	0.801

## Data Availability

The datasets generated and analyzed during the current study and the Nepali version of the SCIM-SR are available from the corresponding author on reasonable request.
